# Fabrication of Miniaturized Paper-Based Microfluidic Devices (MicroPADs)

**DOI:** 10.1038/s41598-018-37029-0

**Published:** 2019-01-09

**Authors:** E. Brandon Strong, Spencer A. Schultz, Andres W. Martinez, Nathaniel W. Martinez

**Affiliations:** 1 Department of Biological Sciences, California Polytechnic State University, San Luis Obispo, CA 93407 USA; 2 Department of Chemistry & Biochemistry, California Polytechnic State University, San Luis Obispo, CA 93407 USA

## Abstract

Microfluidic paper-based analytical devices (microPADs) are emerging as cost-effective and portable platforms for point-of-care assays. A fundamental limitation of microPAD fabrication is the imprecise nature of most methods for patterning paper. The present work demonstrates that paper patterned via wax printing can be miniaturized by treating it with periodate to produce higher-resolution, high-fidelity microPADs. The optimal miniaturization parameters were determined by immersing microPADs in various concentrations of aqueous sodium periodate (NaIO_4_) for varying lengths of time. This treatment miniaturized microPADs by up to 80% in surface area, depending on the concentration of periodate and length of the reaction time. By immersing microPADs in 0.5-M NaIO_4_ for 48 hours, devices were miniaturized by 78% in surface area, and this treatment allowed for the fabrication of functional channels with widths as small as 301 µm and hydrophobic barriers with widths as small as 387 µm. The miniaturized devices were shown to be compatible with redox-based colorimetric assays and enzymatic reactions. This miniaturization technique provides a new option for fabricating sub-millimeter-sized features in paper-based fluidic devices without requiring specialized equipment and could enable new capabilities and applications for microPADs.

## Introduction

Since their introduction in 2007, paper-based microfluidic devices (microPADs) have been explored extensively as platforms for point-of-care diagnostic tests and as tools for basic research and teaching^1–10^. MicroPADs have many attractive qualities such as low cost, small size, and the ability to operate without supporting equipment or sources of power^11^. MicroPADs are typically made by patterning paper with hydrophobic inks, using one of several different printing techniques, in order to define hydrophilic channels and test zones bounded by hydrophobic barriers^2,4^. One common limitation to most methods of patterning paper is that the hydrophobic inks tend to diffuse horizontally in the paper and blur the printed patterns, therefore it can be difficult to produce patterns with dimensions smaller than 1 mm^12^. The ability to fabricate devices with higher-resolution patterns could enable new capabilities for microPADs, as this would allow for the fabrication of smaller devices with higher channel density, which in turn could process smaller volumes of sample in shorter amounts of time. In this article, we describe a new approach for preparing microPADs with higher-resolution features by miniaturizing lower-resolution, wax-printed microPADs.

The concept of shrinking materials in order to fabricate small devices and structures has been explored most famously by the Khine group^13–15^. They used Shrinky-Dinks and other thermoplastic shrink films, which shrink up to 95% in surface area when exposed to heat, to fabricate plastic or polymer-based microfluidic devices as well as other microstructures and metallic nanostructures^15^. Hydrogels, which can shrink upon drying or in response to changes in environmental conditions like pH or temperature, have also been used to fabricate small structures and patterns^16,17^. The advantage of using shrinkable materials for the fabrication of small structures is that it is relatively easy to pattern or fabricate larger, lower-resolution structures, which can subsequently be converted into smaller, higher-resolution structures upon shrinking, without the need for sophisticated microfabrication equipment.

Paper, defined traditionally as a thin sheet made from pressed cellulose fibers^3^, is not commonly thought of as a material that shrinks – even though we probably all have some experience with shrinking cotton cloth, another cellulose-based material, when doing laundry^18,19^. However, we have identified two methods for shrinking paper. The first method involved multiple cycles of soaking in liquid ammonia followed by drying^20^. This approach was used to shrink a dollar bill by ~55% in surface area – the bill shrank anisotropically in plane by ~38% in length and ~28% in width^20^. We did not investigate this method due to the risks of working with liquid ammonia, as well as reduced miniaturization effects as compared to the second method, soaking paper in aqueous solutions of periodate^21,22^, which we optimized for miniaturizing microPADs.

Periodate oxidation of cellulose via the Malaprade reaction has been investigated previously in the context of producing derivatives of cellulose^23–30^ and as a method for covalently linking molecules to the surface of paper^31–34^. The earliest reference to the shrinkage of paper upon exposure to periodate that we could find states that filter paper could be shrunk to 25% of its original surface area (*i.e*., by 75% in surface area) by exposing it to multiple cycles of 0.271-M periodic acid in water for 37 days^21^. The shrinkage of paper was later attributed to a reorganization of the oxidized cellulose chains into non-linear conformations that led to buckling and ultimately to shrinking of the oxidized cellulose fibers^28^. We recently explored the miniaturization of a range of paper types via periodate oxidation and found that all cellulose-based paper types shrink by 60–80% in surface area following saturation in 0.5-M NaIO_4_ for 48 hours^22^. Periodate oxidation has also been shown to shrink cotton cloth and cotton string^35^, but has not, to our knowledge, been investigated previously for the purpose of microPAD fabrication.

Wax printing is one of the most common techniques for patterning paper to fabricate microPADs^36–38^. In this approach, wax is printed onto paper using a solid-ink printer, and then the paper is heated to reflow the wax so that it seeps into the paper and creates a hydrophobic barrier^36^. One limitation of wax printing is the relatively low resolution of the technique, a result of the wax boundaries spreading laterally as well as vertically during the heating step^12^. There is one example of using wax printing to produce high-resolution, sub-millimeter patterns, which was achieved by Tenda *et al*. by printing wax on both sides of the paper followed by a brief heating step using a thermal laminator^12^. Two other techniques for producing sub-millimeter-scale patterns in paper rely on photolithography and laser cutting, respectively^39,40^. To fabricate our high-resolution microPADs, we first optimized the chemical reaction (periodate oxidation) required for miniaturization, we then characterized the miniaturized devices, and, finally, we demonstrated some of the potential advantages and applications of this new type of paper-based device.

## Methods

### Standard MicroPAD Fabrication

Standard microPADs were fabricated via wax printing^36^. The patterns for the devices were designed in Adobe Illustrator (CS6) and printed onto Whatman No. 1 CHR chromatography paper using a solid ink printer (Xerox Phaser 8650). After printing, the sheets of paper were heated for 2 minutes in a convection oven (MTI corporation, Compact Forced Air Convection Oven) set to 195 °C. The devices were then cooled to room temperature, cut out with scissors, and stored under ambient conditions until used.

### Optimization of MicroPAD Miniaturization

Solutions of sodium periodate (NaIO_4_) with concentrations of 0.1, 0.2, 0.3, 0.4, 0.5 and 1.0 M were prepared in deionized (DI) water. The solubility of NaIO_4_ in DI water at room temperature was found to be approximately 0.5 M, and the 1.0-M solution that was prepared was a saturated solution containing solid NaIO_4_. Standard microPADs with dimensions of 4.50 × 4.50 cm were immersed in 25 mL of each periodate solution at room temperature in a covered glass Petri dish. The Petri dishes were shielded from ambient light during the reaction. Devices were removed from the periodate solution after a given reaction time ranging from 6 to 96 hours. The devices were then washed by placing them in a bath of deionized (DI) water for 15 minutes with rocking. After washing, the devices were dried for one hour in a slab gel dryer (Bio-Rad Model 443) at 60 °C and 300 torr. The miniaturized devices were measured with a ruler.

The effect of the wax patterns on the miniaturization process was studied by miniaturizing microPADs with a full wax background, microPADs with wax-outlined channels, and paper with no wax patterns in 0.5-M NaIO_4_ for various time intervals up to 96 hours (diagrams of microPAD types are displayed in Fig. 3B). The devices were washed, dried, and measured as described previously.

The minimum volume of NaIO_4_ solution required for miniaturization was determined by miniaturizing standard microPADs in varying amounts (2–10 mL in 1 mL increments) of 0.5-M NaIO_4_ for 48 hours.

A detailed step-by-step description of the procedure for preparing miniaturized microPADs is provided in the electronic supplementary information.

### Characterization of Miniaturized MicroPADs

**Figure 1:**
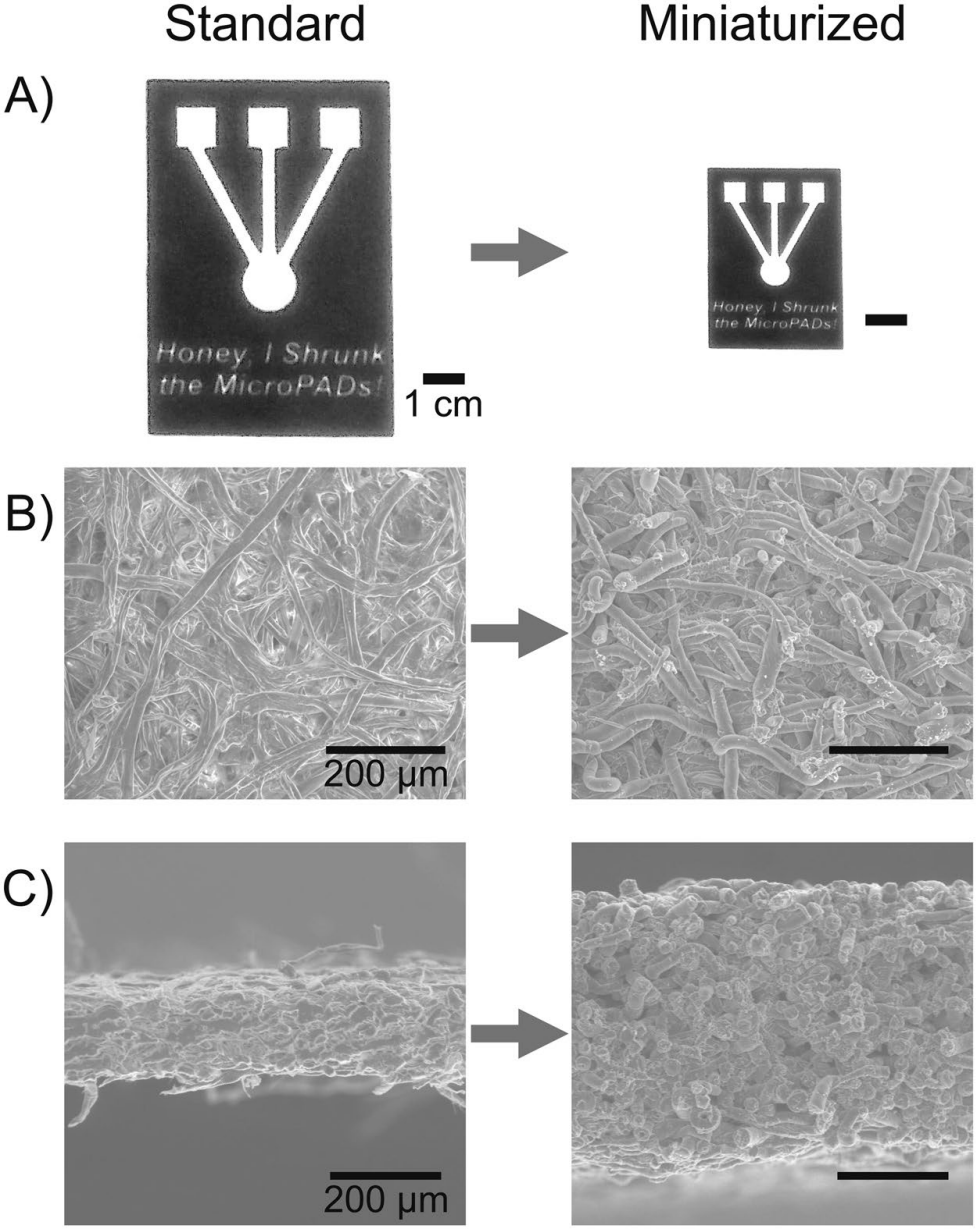
Comparison of microPADs pre- and post-miniaturization. (**A**) Photograph of a wax-printed microPAD displaying high fidelity of the miniaturization process. (**B**) Scanning electron microscope (SEM) surface images of Whatman No. 1 chromatography (CHR) paper (400X magnification). Fibers appear to be more compact following miniaturization. (**C**) SEM cross-sectional images of Whatman No. 1 CHR paper (200X magnification). Miniaturized microPADs displayed a 166% increase in cross-sectional height (thickness). Part C reprinted by permission from Springer Nature, *Cellulose*^22^, 2018.

The surface and cross-section of pieces of chromatography paper and miniaturized chromatography paper (with no wax patterns) were imaged with a scanning electron microscope (SEM, FEI Quanta 200). The height (thickness) of each piece of paper was determined from the SEM images (Fig. 1).

**Figure 2:**
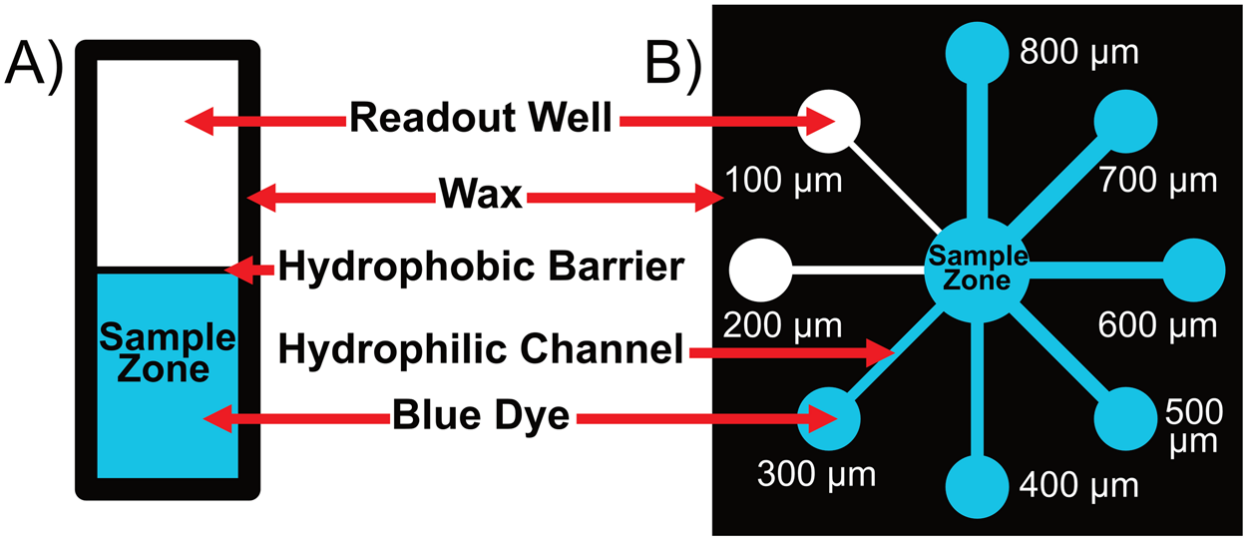
Characterization of miniaturized microPADs. (**A**) Diagram of the device used for the minimum hydrophobic barrier test. A functional barrier prevented fluid from wicking into the empty zone (readout well) for at least 30 minutes. (**B**) Diagram of the device used for the minimum hydrophilic channel test. A channel was determined to be functional if fluid could wick from the sample zone to the readout well.

The minimum functional hydrophobic barrier width and minimum functional hydrophilic channel width were determined for both standard and miniaturized microPADs. A functional hydrophobic barrier was defined as a barrier that prevented aqueous colored dye from wicking across it for at least 30 minutes, and a functional hydrophilic channel was defined as a 5-mm-long channel that could wick aqueous colored dye from a fluid reservoir to a test zone^12^. To determine the minimum functional hydrophobic barrier width, a series of barriers with varying widths (designed in Adobe Illustrator with dimensions in the range of 100–800 µm) were fabricated and then tested by adding 10 µL of an aqueous colored dye solution (either 1-mM Erioglaucine blue dye or 5-mM Allura Red dye in DI water) to one side of the barrier, while looking for evidence of passage of fluid or leakage on the other side of the barrier after 30 minutes (Fig. 2A). The final barrier widths were measured using a dissecting microscope (400X magnification) equipped with a digital camera and a stage micrometer.

To determine the minimum functional hydrophilic channel width, a series of channels with varying widths (designed in Adobe Illustrator with dimensions in the range of 500–1200 µm) were fabricated and then tested by adding 20 µL of aqueous dye to a fluid reservoir on one side of the channel and monitoring passage of the fluid to a test zone on the opposite side of the channel (Fig. 2B). Final channel widths were also measured using a dissecting microscope.

The average wicking velocity was determined for both standard and miniaturized microPADs by adding 15 µL of aqueous dye to a sample zone leading into a channel (1.5 mm in width, 10 mm in length) and measuring the time required for the fluid to wick across the channel. The average wicking velocity was calculated by dividing the length of the channel by the wicking time.

The minimum volume of fluid required for wicking across a 5-mm-long channel for both standard and miniaturized microPADs was measured. The determined minimum functional hydrophilic channel widths for each type of device were used (standard device: 0.6 mm, miniaturized device: 0.3 mm). A range of fluid volumes (0.5–10 µL in 0.5 µL increments) were added to the channels, and the minimum amount of fluid required to fill the channels was recorded.

Geometric fidelity of the miniaturization process was examined by miniaturizing microPADs with varied channel orientations. Channel length was then measured following miniaturization, and the effect of orientation on length was compared via ANOVA (JMP 12.1). A full sheet of Whatman no. 1 chromatography paper (200 mm^2^, with a wax printed 2 cm^2^ grid) was also miniaturized.

### Confirmation of Miniaturized MicroPAD Functionality

#### Glucose Assay

Following miniaturization, functionality as a platform for performing chemical assays was confirmed by performing a glucose assay on a miniaturized microPAD with a sample zone, a reagent zone, a test zone and a waste zone all connected in series by a straight channel (Fig. S1)^41^. The reagents for the assay were deposited onto the reagent zone using a reagent pencil, which was fabricated by pressing a mixture of 66.6% w/w polyethylene glycol (M_n_ 2000 g/mol), 22.2% w/w graphite powder, 0.75% w/w glucose oxidase (GOx, 266 U/mg), 0.52% w/w horseradish peroxidase (HRP, 293 U/mg), and 10.0% w/w 2,2′-azino-bis(3-ethylbenzothiazoline-6-sulphonic acid) (ABTS) into the shape of a cylindrical pellet with a diameter of 3.2 mm using a manual pellet press (Parr Instrument Company)^41,42^. Glucose solutions (3.5 µL) prepared in 1X PBS with concentrations of 0, 0.3, 0.6, 0.9 and 1.2 mM were applied to the sample zone of the device and a colorimetric readout was generated in the test zone. The intensity of the color produced in the test zones was measured via digital image colorimetry (DIC)^43^, where the mean color intensity in the red channel of the test zones was measured using a smartphone (Samsung Galaxy Note 4) and the Color Grab application^9^.

#### Enzyme viability

Solutions of horseradish peroxidase (HRP, 0.6–10.5 U/mL in 1XPBS, 2 µL) were added to circular test zones (5.5 mm in diameter) on miniaturized microPADs. Immediately after drying the HRP solutions on the devices under ambient conditions, 3 µL of tetramethylbenzidine liquid substrate (TMB, Sigma Aldrich, T4444) was added to each test zone, and the reaction was allowed to proceed for 20 minutes. Sulfuric acid solution (H_2_SO_4_, 1 M in DI water, 2 µL) was added to each test zone to quench the reaction, and the test zones were dried under ambient conditions. The mean intensity of each zone was measured via DIC as described previously.

## Results and Discussion

Upon reacting with aqueous periodate, the microPADs shrunk in the plane of the paper (Fig. 1A), while the height (thickness) of the paper increased (Fig. 1C), which is analogous to what is observed when shrinking thermoplastic films^13^. The wax patterns shrunk proportionally with the paper resulting in high-fidelity, miniaturized reproductions of the original standard devices (Fig. 1A). SEM images comparing a piece of untreated chromatography paper to a piece of miniaturized chromatography paper show that the paper fibers appear to swell and pack more densely during the miniaturization process (Fig. 1B,C). The images also suggest that the size of the pores in miniaturized paper are smaller compared to the original paper. The increased density of cellulose fibers in the miniaturized devices, resulted in greater rigidity of the device, allowing for easier manipulation of the diagnostic platform.

**Figure 3:**
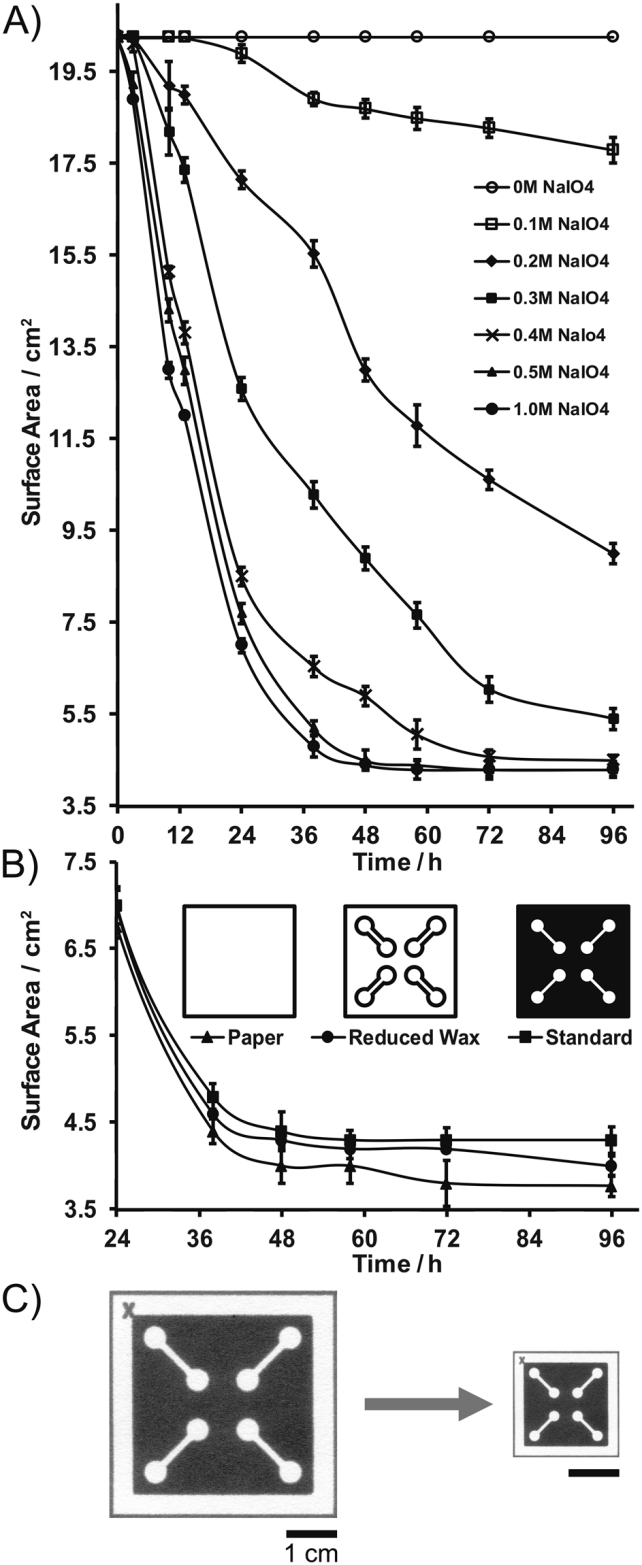
Miniaturization of microPADs over time. (**A**) Plot of microPAD surface area versus reaction time for various concentrations of aqueous sodium periodate (NaIO_4_). (**B**) Miniaturization comparisons of: non-patterned chromatography paper, microPADs with channel outlines (reduced wax), and microPADs with a full wax background (standard) in 0.5 M NaIO_4_. Non-patterned chromatography paper displayed the greatest amount of miniaturization, albeit to a minor degree. For both plots, data points represent the mean of three replicates, and error bars represent one standard deviation from the mean. (**C**) Photograph of miniaturized microPAD (~78% reduction in surface area).

The degree of miniaturization of microPADs can be controlled by tuning both the concentration of periodate and the reaction time (Fig. 3A). For example, when 0.1-M periodate was used, the devices shrunk more slowly, and the reaction needed at least 24 hours before any change in size was observed. When 0.5-M periodate was used, some miniaturization was observed after only 6 hours, most of the miniaturization took place within 48 hours, and minimal additional miniaturization was observed after 72 hours. The amount of wax patterning on microPADs had only a small impact on the degree of miniaturization (Fig. 3B). After 96 hours in a 0.5-M periodate solution, full-wax devices shrunk 78.6% in surface area, wax-outlined devices shrunk 79.8% in surface area, and paper without wax patterns shrunk 80.9% in surface area. One possible explanation for these results is that the wax patterns protected some of the cellulose molecules from reacting completely with the periodate, which may have slightly limited the degree of miniaturization in the case of the full-wax devices.

Since our goal with this project was to establish a method for miniaturizing microPADs, we selected 0.5-M periodate and 48 hours of reaction time for the optimized miniaturization procedure (Fig. 3A). Higher concentrations of periodate cannot be achieved due to the solubility of NaIO_4_ in water at room temperature, and a saturated solution of periodate (e.g., 1.0 M) did not shrink the devices any further or faster than the 0.5-M solution. Longer reaction times than 48 hours did not result in significant additional miniaturization either. Devices that were miniaturized for 72 or 96 hours were only 0.5% smaller than devices miniaturized for 48 hours (Fig. 3).

After reacting in 0.5-M periodate for 48 hours, the average reduction in size for a standard microPAD was 78% in surface area, or 53% in linear dimensions (Fig. 3, Table 1). To achieve this level of miniaturization, a minimum of 0.3 mL of 0.5-M periodate solution per cm^2^ of microPAD surface area was required (Fig. S2). When lower volumes of solution were used, the devices did not shrink to the same extent. Additional solution, above 0.3 ml/cm^2^, had no effect on the miniaturization process, therefore we recommend using a minimum of 0.4 ml/cm^2^ of the periodate solution to ensure proper miniaturization, as well as complete submersion of the devices.

**Table 1:** Summary data table comparing standard versus miniaturized microPADs. Miniaturized microPADs displayed a significant reduction in all characteristics except cross-sectional width.

Characteristic	Replicates	Standard MicroPAD	Miniaturized MicroPAD	% Change
Surface Area (cm^2^)	50	20.25	4.41 ± 0.11	−78.3%
Linear Dimension (cm)	50	4.50	2.10 ± 0.03	−53.3%
Cross-Sectional Height (µm)	1	188	500	166.0%
Minimum Hydrophilic Channel (µm)	14	585 ± 54	301 ± 42	−48.5%
Minimum Hydrophobic Barrier (µm)	10	550 ± 37	387 ± 21	−29.6%
Average Wicking Velocity (mm/s)	11	0.480 ± 0.076	0.235 ± 0.017	−51.0%
Minimum Volume (µL)	10	8.0 ± 1.2	2.0 ± 0.2	−75.0%

Values are given as the mean of all replicate measurements, +/− one standard deviation of the mean.

For miniaturized microPADs, the narrowest functional hydrophobic barrier had an average width of 387 ± 21 µm (Fig. 2A, Table 1), while the narrowest functional hydrophilic channel had an average width of 301 ± 42 (Fig. 2B, Table 1). For comparison, the narrowest hydrophobic barrier in a standard microPAD had an average width of 550 ± 37 µm, and the narrowest hydrophilic channel had an average width of 585 ± 54 µm (Table 1). The differences represent a 30% reduction in the width of the smallest hydrophobic barriers and a 49% reduction in the width of the smallest hydrophilic channels for a miniaturized microPAD as compared to a standard microPAD. The ability to fabricate microPADs with smaller, higher-resolution features should allow for higher channel density to be incorporated into microPADs. For example, based on the determined minimum hydrophilic channel and hydrophobic barrier widths, a 1-cm-wide microPAD could theoretically accommodate up to 8 parallel hydrophilic channels in the case of a standard microPAD, but could accommodate up to 14 parallel hydrophilic channels in the case of a miniaturized microPAD. Compared to the method published by Tenda *et al*., which reported a minimum hydrophobic barrier width of 467 ± 33 µm and a minimum hydrophilic channel width of 228 ± 33 µm^12^, our method allows for the fabrication of smaller hydrophobic barriers but slightly larger hydrophilic channels. An interesting observation is that the two methods of fabrication are orthogonal and could potentially be combined to fabricate devices with even smaller channels and barriers than could be achieved using either method independently.

The average wicking velocity in miniaturized microPADs was reduced by a factor of ~2 compared to standard devices (Table 1). Fluid wicked across channels (1.5 mm in width × 10 mm in length) in miniaturized devices in 42 ± 3 s, for an average rate of 0.24 ± 0.02 mm/s, while fluid wicked across channels with the same dimensions in standard devices in 21 ± 4 s, for an average rate of 0.48 ± 0.08 mm/s. The decrease in average wicking velocity can likely be attributed to a combination of two factors: a decrease in the effective pore size and an increase in hydrophobicity of the miniaturized paper. When shrinking paper, the cellulose fibers contract and pack more tightly, which, in turn, leads to smaller spaces between the fibers, as was observed by SEM (Fig. 1B,C). Smaller pores would be expected to slow down wicking as predicted by the Lucas-Washburn model^44–47^. Periodate oxidation of paper also reduces the number of hydroxyl groups on paper, which would increase the hydrophobicity of the resulting material compared to untreated paper and also contribute to slower wicking. Slower wicking will not necessarily impact the performance of miniaturized devices since these devices would typically be smaller than standard microPADs, so the fluid would be wicking over shorter distances. Slower wicking rates could also allow for increased assay sensitivity by increasing reaction time within channels and test zones. Future microPADs could also potentially incorporate both standard and miniaturized paper in multi-layered devices to harness the advantages of both materials.

The volume of fluid required to fill the device was also reduced in miniaturized microPADs. We found that a miniaturized microPAD required 2 µL to fill a 5-mm-long channel, while a standard microPAD required 8 µL to fill a channel of the same length (Table 1). The reduction in volume of fluid can be attributed to two effects. First, smaller channels can be fabricated in miniaturized microPADs, therefore these channels will require less fluid. In our experiment, the width of the channel in the miniaturized microPAD was 0.3 mm and the width of the channel in the standard microPAD was 0.6 mm. Second, because the fibers in the miniaturized devices are packed more tightly, there is less void space in the miniaturized devices that can fill with fluid. In general, lower volume requirements are favorable since they allow for assays to be performed on smaller sample sizes, and these devices require smaller quantities of deposited reagents.

**Figure 4:**
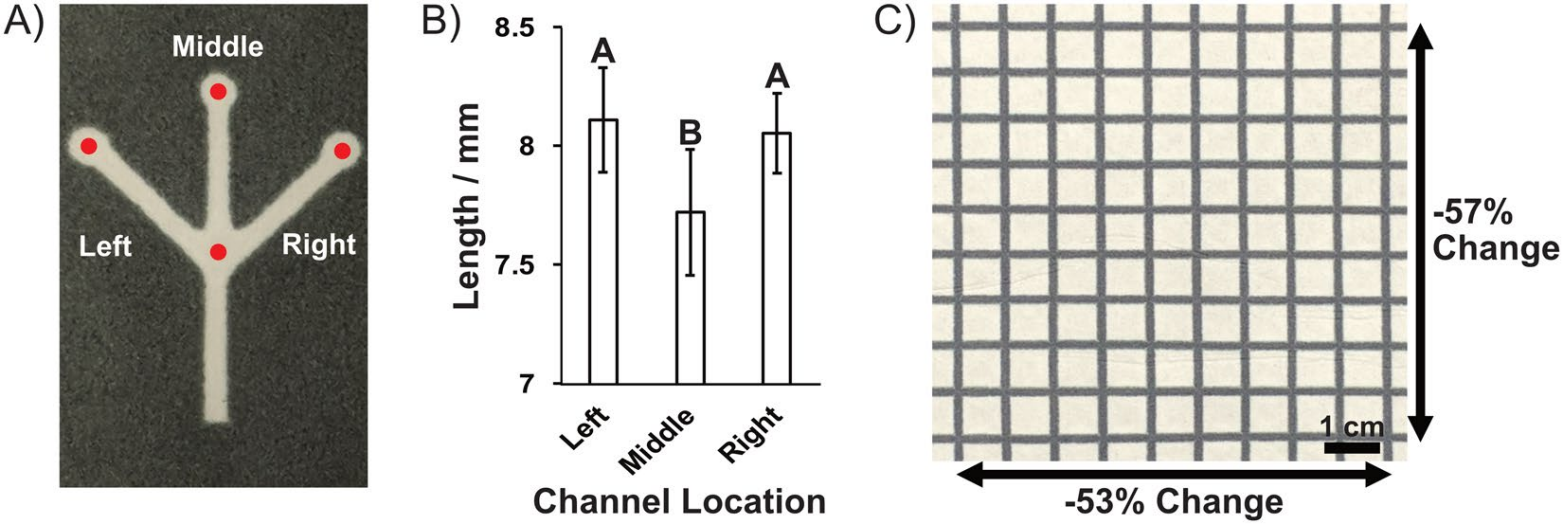
Examination of the geometric fidelity of the miniaturization process. (**A**) Photograph of miniaturized microPAD (0.5-M, 48 hours). Left, middle, and right channels all initially 16 mm in length prior to miniaturization. Red dots indicate point of measurement. (**B**) Graph of left, middle, and right channel length (photograph in part A) following miniaturization (n = 9). There was no significant difference between left and right channels (p = 0.855), but the middle channel was significantly shorter than both the left (p = 0.003) and right (p = 0.010) channels. Error bars represent one standard deviation from the mean. (**C**) Photograph of miniaturized grid on a full-sized sheet of 200 mm^2^ Whatman no. 1 chromatography paper. As expected, the paper shrank anisotropically^22^, but displayed good miniaturization fidelity across the sheet.

A final important characteristic of miniaturized microPADs was the geometric fidelity of the devices following miniaturization. MicroPADs displayed differing levels of channel miniaturization dependent upon orientation (Fig. 4A,B). This was to be expected due to the anisotropic nature of the miniaturization process^22^. While the difference in channel lengths was minimal (Fig. 4B), and therefore should have minimal effect on microPAD functionality, microPADs could be designed with parallel channels to promote fidelity, or the channel design could be adjusted to account for the anisotropy of the miniaturization. A full sheet of Whatman no. 1 chromatography paper (200 mm^2^, initial 2 cm^2^ grid) also displayed high fidelity following miniaturization (Fig. 4C). This indicates that full sheets of microPADs could successfully be fabricated via this process.

**Figure 5:**
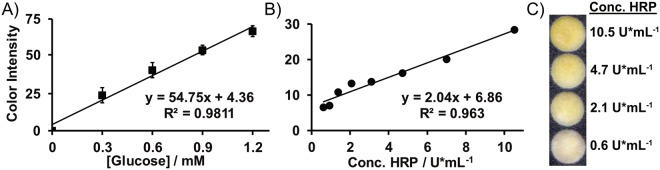
Miniaturized microPADs as platforms for biochemical assays. (**A**) Calibration curve as generated from a colorimetric paper-based glucose assay. The data was fit with a linear trendline. Data points represent the mean of eight replicates and error bars represent one standard deviation of the mean. (**B**) Calibration curve for a colorimetric horseradish peroxidase (HRP) assay. The data was fit with a linear trendline. Data points represent a single replicate. (**C**) Photograph of the test zones after performing the HRP assays. Higher HRP concentrations produced higher color intensities in the test zones.

The performance of miniaturized microPADs as platforms for biochemical assays was confirmed by performing a glucose assay (Fig. 5A). The results were quantified via digital image colorimetry and were used to generate a linear calibration curve with a high R^2^ value (0.98). In addition to analytical performance, the glucose assay confirmed enzyme functionality on miniaturized microPADs as both glucose oxidase and horseradish peroxidase (HRP) activity are required for the assay. Furthermore, since this assay relies on redox chemistry, it demonstrated that the periodate was either completely removed from the miniaturized devices during the wash step or that any residual periodate did not interfere with the assay.

The viability of enzymes on oxidized cellulose fibers was also confirmed by performing a colorimetric assay for HRP on miniaturized devices. After drying the HRP solutions on the devices, a concentration-dependent color intensity was produced upon addition of the substrate for the enzyme (Fig. 5B,C). This result is significant given that several diagnostic assays rely on the activity of enzymes for signal amplification or for direct detection of analytes^48^.

## Conclusions

We developed a new method for fabricating higher-resolution microPADs by shrinking wax-patterned devices. The method does not require any specialized equipment and can be used readily by any researcher working with paper-based devices. We believe that the ability to miniaturize microPADs will enable new capabilities and applications for this class of devices. For example, miniaturized devices can incorporate higher channel density compared to standard microPADs and can be used as platforms for the same types of biochemical assays that are typically performed on standard microPADs. The miniaturized devices also require smaller volumes of sample per unit surface area of the device and require smaller quantities of deposited reagents. Furthermore, the miniaturized devices possess increased rigidity, allowing for easier manipulation of the diagnostic platform.

The method for shrinking microPADs is highly tunable and can be controlled easily by changing the concentration of periodate or the reaction time. This method could also be readily applied and adapted toward the fabrication of other types of devices, structures or cellulose containing materials. Taken together, this novel method of device fabrication is a significant step towards the development of a new generation of paper-based microfluidic devices for which we are currently exploring additional applications.

## Supplementary information

Electronic Supplemental Information for Fabrication of Miniaturized Paper-Based Microfluidic Devices (MicroPADs)

## Data Availability

The datasets generated during and/or analyzed during the current study are available from the corresponding author on reasonable request.
