# Supplementary material for: Fabrication of Miniaturized Paper-Based Microfluidic Devices (MicroPADs)
Source: Sci Rep. 2019 Jan 9;9:7. doi: 10.1038/s41598-018-37029-0 (PMC6327054; doi:10.1038/s41598-018-37029-0)
Supplement: Supplementary file 1 — Electronic Supplemental Information for Fabrication of Miniaturized Paper-Based Microfluidic Devices (MicroPADs) [file 41598_2018_37029_MOESM1_ESM.pdf]

SUPPLEMENTARY INFORMATION FOR:

Fabrication of Miniaturized Paper-Based Microfluidic Devices (MicroPADs)

E. Brandon Strong[1], Spencer A. Schultz[2], Andres W. Martinez[2],

and Nathaniel W. Martinez*[1]

[1]Department of Biological Sciences, [2]Department of Chemistry & Biochemistry, California Polytechnic State University, San Luis Obispo, CA 93407

* Corresponding author E-mail: nmarti32@calpoly.edu

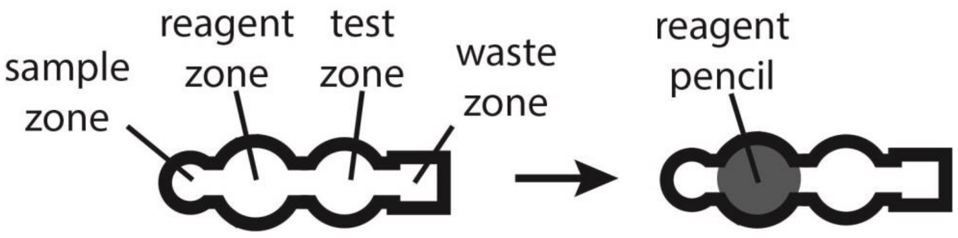

Figure S1. Diagram of microPAD used for the glucose assay.

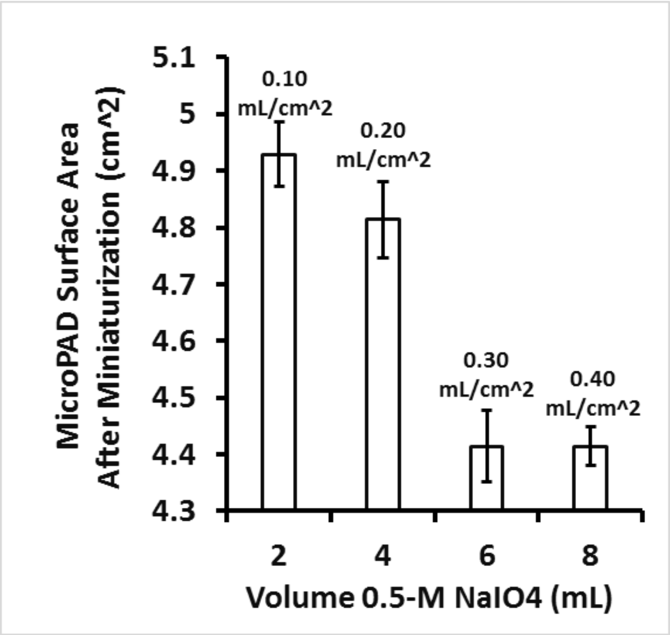

Figure S2. Bar graph of miniaturized microPAD surface area versus volume of 0.5-M $NaIO_4$ used for the miniaturization process. The initail surface area of all devices was 20.25 cm^2. The height of the bars represent the mean of seven replicates and the error bars represent one standard deviation from the mean. These results indicate that a minimum of 0.30 mL 0.5-M $NaIO_4$ per cm^2 of microPAD is required for complete miniaturization.

STEP-BY-STEP FABRICATION OF MINIATURIZED MICROPADS

1. Design microPADs using a drawing program (e.g., Adobe Illustrator). When designing the devices, it is important to account for lateral wax diffusion during heating and the subsequent miniaturization. Printed wax barriers will diffuse ~275 µm in every direction in the plane of the paper upon heating, followed by an ~53% reduction in linear dimensions upon miniaturization.
2. Print the designs onto chromatography paper (CHR 1) using a solid-ink printer (e.g., Xerox Phaser 8560).
3. Heat the printed pages using a forced air convection oven (or other heat source, such as a hot plate) set to 195 $^{\circ}$C for 2 min.
4. Prepare a 0.5-M solution of sodium periodate in deionized water (Note: periodate is a strong oxidizer and should be handled with care. Please refer to the SDS for sodium periodate for information on proper handling of this reagent). At least 0.4 mL of solution per cm^2 of microPAD surface area should be prepared. Seeing as the devices must be fully submerged, excess periodate solution is recommended.
5. Submerge the devices in the periodate solution in an appropriately-sized container made from glass or plastic. Cover the container and place in a dark area and allow the reaction to proceed for 48 hours.
6. Remove the miniaturized microPADs from the periodate solution and wash them in deionized water for 15 minutes. Placing the water bath on a rocker will promote water flow over the devices. Failure to properly wash any remaining periodate out of the paper will lead to yellowing and increased hydrophobicity of the paper. Dispose of the remaining periodate solution in an appropriate waste container.

7. Place the devices in between two pieces of blotting paper in a gel slab dryer for 1 h (60 ^{o}C, 300 torr). If a slab dryer is unavailable, devices can also be dried between two pieces of blotting paper under physical pressure (e.g. between the pages of a large book, chemistry or biology textbooks are particularly effective) overnight.
